# A Method of Damage Detection Efficiency Enhancement of PZT Sensor Networks under Influence of Environmental and Operational Conditions

**DOI:** 10.3390/s23010369

**Published:** 2022-12-29

**Authors:** Michal Dziendzikowski, Mateusz Heesch, Jakub Gorski, Kamil Kowalczyk, Krzysztof Dragan, Ziemowit Dworakowski

**Affiliations:** 1 Airworthiness Division, Air Force Institute of Technology, ul. Ks. Boleslawa 6, 01-494 Warszawa, Poland; 2 Department of Robotics and Mechatronics, Faculty of Mechanical Engineering and Robotics, AGH University of Science and Technology, 30-059 Krakow, Poland

**Keywords:** structural health monitoring, damage detection capabilities enhancement, environmental and operational conditions compensation, PZT transducers applications

## Abstract

Two performance parameters are particularly important for the assessment of structural health monitoring (SHM) systems, i.e., their damage detection capabilities and risk of false positive indications due to varying environmental and operational conditions (EOCs). A reduced ratio of false-positive indications can be of significant importance for particular applications, for example, in aerospace, where the costs of unplanned maintenance procedures can be very high. In such cases, the reduction of the false calls ratio can be critical for the possibility of the practical application of the system, apart from damage detection efficiency and system costs. Among various sensor technologies, PZT networks are proven to be one of the most universal approaches to SHM, and they were successfully applied in different scenarios. Moreover, many EOCs which may have an impact on the risk of false positive indications have been identified. Over the years, different approaches to the influence of EOCs compensation have been proposed. Compensation methods can be tailored to the particular way in which a given measurement condition, for example, ambient temperature, alters signals acquired by the PZT network or can be formulated to be also applied in the more general case. In the paper, a method for enhancement of damage detection efficiency under influence of EOCs of general nature is proposed. The particular measurement condition affecting signals acquired by PZT sensors neither needs to be measured, which could be hard in some cases, but also nor even have to be identified. The efficiency of the proposed compensation algorithms is verified based on the example of experimental results obtained under varying temperatures.

## 1. Introduction

Structural health monitoring (SHM) technologies [1,2,3,4,5] are important for further advance of novel approaches to industry and transport organization known as Industry 4.0 paradigm [6] providing an on-line evaluation of structures safety which can be further used for autonomous control and optimization of industrial processes, for example, by artificial intelligence. One of the most fruitful approaches to SHM is based on guided wave excitation by a network of PZT transducers. In the so-called pitch–catch framework, a bundle of signals is collected for a given measurement. For every pair of transducers g–s of the network, elastic waves actuated by the transducer g are received by the transducer s, providing a signal, usually the voltage on s. Repeating the procedure for all sensing paths g–s of the network completes the measurement process, and the health of the structure can be evaluated. For the purpose of damage detection and structure assessment, the so-called damage indices (DIs) can be calculated. Damage indices are signal characteristics, which are defined using reference signals acquired for the initial state of the structure—the so-called baselines [7,8]. Depending on the adopted approach, DIs can be either finely tuned to be sensitive to signal changes proper for a given damage type location, or they can carry limited information about the signal, for example, peak values, RMS, or other integral characteristics. PZT ceramic sensors have proven to be the technology of the universal application capabilities [3,7,8,9,10]. In particular, it was successfully applied to cracks detection, and their growth monitoring [11,12], bolt and rivet joints monitoring [13,14,15], corrosion detection [16,17], civil infrastructure monitoring [18,19], and many other applications [20].

For successful PZT network application, it is important to consider also the risk of false positive indications under varying environmental and operating conditions (EOCs) since the high false call ratio of an SHM system can prevail over its benefits in some cases. Changes in measurement conditions can alter the acquired signals, which may cause non-damage-related changes in damage index values within the network. Therefore, if a given measurement condition was not taken into account in the damage index definition, for example, by a proper collection of baseline signals database, it could lead to a false positive indication of the SHM system. A lot of research has been devoted to the investigation of the influence of different EOCs on the damage detection capability of PZT sensors as well as the design of efficient methods of EOCs compensation [21]. Among various conditions which may have an impact on signals acquired by PZT sensors are, in particular: the temperature [22,23,24,25,26,27], operational loads [28,29,30,31], bonding defects and adhesive layer thickness [32,33,34]. For EOCs impact compensation, different strategies can be implemented. Some of the methods developed for external measurement conditions compensation for SHM systems based on PZT networks are based on particular effects a given condition can have on the acquired signals. Then, by a proper signal transformation as described in [23,24,35] or design of damage index in the way that it is insensitive to expected alteration of signal due to measurement condition change as in [36,37], the undesired influence of EOCs can be diminished. In such approaches, first, it is needed to verify how a given condition can alter the acquired signals. Therefore, to apply such an approach in practice, it is required to evaluate all the effects which may have an impact on the properties of PZT sensors and design appropriate algorithms for signal transformation. For proper algorithm definitions, it is important to reproduce measurement conditions in a repeatable manner to verify the characteristics of signal behavior under varying conditions. Moreover, signal compensation algorithms can be parametric; therefore, it is necessary to provide a measurement system for all relevant factors, which in general, can be non-homogeneously distributed over the network. For controllable and measurable factors, for example, temperature, external forces applied to the structure or humidity; such requirements can be easily satisfied. However, there are other important conditions that can contribute to signal alteration but are much harder to be measured or represented in a repeatable manner, for example, the strength of transducers bonding with the structure or aging effects of PZT ceramic. In addition, it is not clear if different algorithms for signal compensation can be applied mutually and commutatively if two or more measurement conditions are changed.

Another common approach, which can be used in the general case, is the proper definition of baseline signals database. In the procedure called Optimal Baseline Selection (OBS) [23], prior to the calculation of damage indices used for structure assessment, the database of reference signals is verified to find the best matching signal based on some similarity measure. It is assumed that baselines in the database are acquired for the pristine state of the structure for a broad spectrum of operational conditions. While it is a general nonparametric procedure that can be applied to any environmental or operational condition, acquisition of a representative database for real structures can sometimes be difficult to obtain [21] and can negatively impact damage detection capabilities of the system [37]. In [37], damage indices compensation based on a median value obtained for all sensing paths of the network was proposed. Similarly, as in the case of OBS, the method can be applied in general; however, it has limited applicability when environmental condition is not homogeneous across the network. Moreover, the application of this method reduces the information content of the acquired data to a single damage index value calculated for the entire PZT network; therefore, damage localization methods based on DIs distribution across the network, for example, RAPID algorithm [38] or its modifications [39] cannot be used.

In this paper, the generalization of the damage indices compensation algorithm presented in [40] is proposed. The method was designed to be applied for general measurement conditions, also in the nonhomogeneous case. The main idea presented in the paper was to use sensing paths of the network which are not influenced by damage as a basis to estimate the impact of external measurement conditions on damage indices obtained for sensors constituting other sensing paths of the network. The main advantage of the proposed damage indices compensation methods is that EOCs driving undesirable effects neither need to be measured, which could be hard in some cases, but also nor even have to be identified. It is the damage index itself which, through its values obtained over all sensing paths of the network, carries joint information about factors influencing measurement outcome, and a proper combination of damage index values is used for compensation. In [40] original algorithm method was used in the case of aging effects compensation as well as in the case of sensor malfunction. In this study, the efficiency of different schemes for damage indices compensation in a broad range of temperature variations, as well as its nonhomogeneous distribution, is investigated.

The paper is organized as follows. In the following section, the approach to damage indices compensation is defined and clarified. In Section 3, the description of the experiment is delivered. The subsequent section provides a discussion of the obtained results, then the paper is summarized.

## 2. Definition of Damage Indices Compensation Methods

In this section definition of different compensation formulas used in this paper is provided. damage indices compensation schemes are formulated in Equations (13)–(15). While those definitions can be used and justified on their own, the main assumptions and derivation steps of the original method presented in [40] are recalled in this paper, for better clarification of the idea.

Let us consider a network of sensors for which for every pair of transducers: i.e., a generator *g* and a sensor *s*, there exist two different reference transducers $r_{g}$, $r_{s}$ for which changes of a given damage index values on sensing paths $g–r_{g}$, $s–r_{s}$, $r_{g}–r_{s}$ are only due to nondamage related measurement conditions, causing the drift effect of DI:

(1) $$\begin{array}{cc} \; & DI\left(g,r_{g}\right)=DI_{drift}\left(\delta_{g},\delta_{r_{g}}\right),\\ \; & DI\left(s,r_{s}\right)=DI_{drift}\left(\delta_{s},\delta_{r_{s}}\right),\\ \; & DI\left(r_{g},r_{s}\right)=DI_{drift}\left(\delta_{r_{g}},\delta_{r_{s}}\right), \end{array}$$

where $\delta_{t}=\left(\delta_{t}^{1},\ldots ,\delta_{t}^{n}\right)$ denotes a set of continuous parameters influencing a given transducer *t* and eventually changing DI values on all sensing paths emerging from *t*.

**Figure 1:**
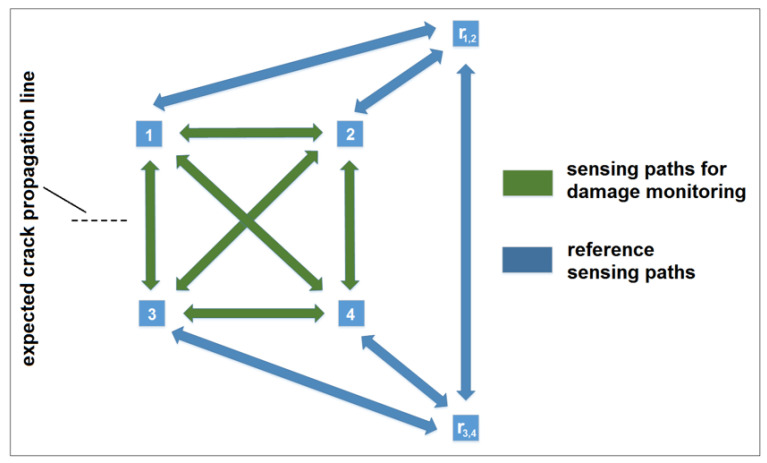
An example of a PZT network containing reference transducers.

A scheme of a network containing reference transducers is shown in the figure below. The network is designed to detect and monitor crack growth which will eventually propagate along the expected propagation line (Figure 1). In this network design, sensing paths 1–3 could be used to detect crack entry into the monitored area covered by the network, whereas sensing paths 1–4, 2–3, and 2–4 would give information about its growth.

Unless the last path is crossed by the crack, i.e., 2–4 and assuming that contribution to DI values due to wave reflection from the crack are negligible for sensing paths $1—r_{1,2}$, $2—r_{1,2}$, $3—r_{3,4}$, $4—r_{3,4}$, and $r_{1,2}–r_{3,4}$ compared to, there should be no contribution from the crack to damage index values obtained for those sensing paths. Therefore, according to the condition given by the Equation (1), transducers $r_{1,2}$, $r_{3,4}$ constitute a reference frame for the subnetwork formed by sensors 1–2–3–4, i.e., for this setup $r_{1,2}$ is a reference transducer for sensors 1 and 2 while $r_{3,4}$ is reference transducer for sensors 3 and 4.

Parameters $\delta_{t}^{i}$, i=1,…,n altering the performance of transducers *t* and driving the DIs drift can be of a very different nature. These can be changes in environmental working conditions of the network, for example, temperature, humidity, or pressure changes, which can be easily measured and compensated by proper calibration, but also these can be changes in piezoelectric properties of transducers or strength of their bonding to the structure, which are very hard to be measured and verified in practice. In [40], linear dependence of damage index for a given sensing path *g*–*s* on both of the effects is assumed:

(2) $$DI\left(g,s\right)=DI_{drift}\left(\delta_{g},\delta_{s}\right)+DI_{damage}\left(g,s\right).$$

The first term describing DI flow depends on a set of factors $\delta_{g},\delta_{s}$ acting on the generator *g* and the sensor *s*, while the second term describes the response of damage index on damage presence. Parameters $\delta_{g},\delta_{s}$ do not need to be observed directly, but their influence on the performance of transducers g and s is revealed in the drift of DI on a given sensing path. For a given transducer *t* parameters $\delta_{t}$ can be scaled in the way that $\delta_{t}^{i}=0$ for i=1,…,n denotes initial working conditions for which baseline signals were collected, therefore:

(3) $$DI_{drift}\left(0,0\right)=0$$

since, ideally, in the absence of damage, the signal acquired on sensing path g–s should be the same as the baseline used to calculate the damage index value. Therefore, in the first order of approximation:

(4) $$DI_{drift}\left(\delta_{g},\delta_{s}\right)\approx \sum_{i=1}^{n}C_{g,i}\delta_{g}^{i}+\sum_{i=1}^{n}C_{s,i}\delta_{s}^{i}$$

where

(5) $$C_{g,i}=\frac{\partial DI_{drift}}{\partial \delta_{g}^{i}}\left(0,0\right),\;C_{s,i}=\frac{\partial DI_{drift}}{\partial \delta_{s}^{i}}\left(0,0\right).$$

Assuming that the network is composed of PZT transducers of the same type and since DIs values should be symmetric with respect to switching between the generator and the sensor [7,8]:

(6) DI(g,s)≈DI(s,g),

then the functional form of drift should be the same for all sensing paths, and only parameter values $\delta_{t}^{i}$, i=1,…,n driving the drift on the transducer *t* can be different across the network. In that case

(7) $$C_{g,i}=C_{s,i}\equiv C_{i}\;\forall g,s$$

and the drift effect of damage index on sensing path *g*–*s* can be estimated by the following combination:

(8) $$DI_{drift}\left(\delta_{g},\delta_{s}\right)\approx DI_{g,r_{g}}+DI_{s,r_{s}}-DI\left(r_{g},r_{s}\right).$$

Indeed, since it is assumed that sensing paths $g–r_{g}$, $s–r_{s}$, $r_{g}–r_{s}$ are not influenced by damage, we have, according to the Equation (4):

(9) $$\begin{array}{cc} DI\left(g,r_{g}\right)+DI\left(s,r_{s}\right)-DI\left(r_{g},r_{s}\right) & =C_{i}\delta_{g}^{i}+C_{i}\delta_{r_{g}}^{i}+C_{i}\delta_{s}^{i}+C_{i}\delta_{r_{s}}^{i}-C_{i}\delta_{r_{g}}^{i}-C_{i}\delta_{r_{s}}^{i}=\\ \; & =DI_{drift}\left(\delta_{g},\delta_{s}\right) \end{array}$$

where Einstein’s summation convention has been used to omit unnecessary summation symbols.

Therefore, for compensated damage index of the form:

(10) $$DI_{ref}^{comp}\left(g,s\right)=DI\left(g,s\right)-DI\left(g,r_{g}\right)-DI\left(s,r_{s}\right)+DI\left(r_{g},r_{s}\right)$$

considering the Equation (2) we have:

(11) $$DI_{ref}^{comp}\left(g,s\right)\approx DI_{damage}\left(g,s\right),$$

thus only damage contributes to the compensated damage index.

The proposed compensation formula given by Equation (10) requires additional transducers in the network to be present, and these cannot be used for damage monitoring in accordance with the assumption given by the Equation (1). In some cases, especially for sparse PZT arrays like the one presented in Figure 1, this can introduce too much redundancy in the system. Moreover, it is not always possible to determine a priori areas of the structure where the probability of damage occurring is very low. Yet it is usually the case, that since guided wave interaction with compact damage is a local phenomenon, then for a given sensor of the network, there should exist sensing paths emerging from it that are not influenced by damage, unless damage emerged very close to the sensor location.

Assuming that the highest damage index values should be obtained for sensing paths which are both influenced by damage and change of measurement conditions, then one could replace drift contributions $DI(g,r_{g})$$DI(s,r_{s})$ coming from the generator *g* and sensor *s* by appropriate estimates, in this paper it is proposed to adopt the following substitutions:

(12) $$DI\left(g,r_{g}\right)\equiv Med\left(M_{k}\left(g\right)\right),\;DI\left(s,r_{s}\right)\equiv Med\left(M_{k}\left(s\right)\right),$$

where $M_{k}\left(g\right)$, $M_{k}\left(s\right)$ are median values of the sets of *k* smallest damage index values obtained for sensing paths emerging from the generator *g* or *s* respectively. In general, the signal on the sensing path between sensors contributing to sets $M_{k}\left(g\right)$ and $M_{k}\left(s\right)$ can be influenced by damage. In the paper, three different alternatives are considered for the replacement of the contribution $DI(r_{g},r_{s})$ to damage index drift estimation as in Equation (10):

- the so-called standard approach, based on proposition presented in [40], for which $DI\left(r_{g},r_{s}\right)\equiv Med\left(M_{k}\right)$ where $Med(M_{k})$ is median of the set of *k* smallest damage index values obtained for the entire PZT network;
- the symmetric approach for which $DI(r_{g},r_{s})$ is estimated symmetrically by drifts contributions corresponding to generator *g* and *s* by the term $\frac{1}{2}Med\left(M_{k}\left(g\right)\right)+\frac{1}{2}Med\left(M_{k}\left(s\right)\right)$;
- the minmax approach for which $DI\left(r_{g},r_{s}\right)\equiv \min\left(Med\left(M_{k}\left(g\right)\right),Med\left(M_{k}\left(s\right)\right)\right)$.

Therefore, in this paper, three compensation formulas for damage indices are proposed:

(13) $$\begin{array}{cc} \; & \;DI_{standard}^{comp}\left(g,s\right)=\max\left(DI\left(g,s\right)-Med\left(M_{k}\left(g\right)\right)-Med\left(M_{k}\left(s\right)\right)+Med\left(M_{k}\right),0\right) \end{array}$$

- for standard approach;

(14) $$\begin{array}{cc} \; & \;DI_{symm}^{comp}\left(g,s\right)=\max\left(DI\left(g,s\right)-\frac{1}{2}Med\left(M_{k}\left(g\right)\right)-\frac{1}{2}Med\left(M_{k}\left(s\right)\right),0\right) \end{array}$$

for symmetric approach;

(15) $$\begin{array}{cc} \; & \;DI_{minmax}^{comp}\left(g,s\right)=\max(DI\left(g,s\right)-Med\left(M_{k}\left(g\right)\right)-Med\left(M_{k}\left(s\right)\right)+\\ \; & \;\;\;\;\;+\min(Med\left(M_{k}\left(g\right)\right),Med\left(M_{k}\left(s\right)\right)),0)=\\ \; & \;\;\;\;\;=\max(DI\left(g,s\right)-\max\left(Med\left(M_{k}\left(g\right)\right),Med\left(M_{k}\left(s\right)\right)\right),0) \end{array}$$

- for minmax approach.

The methods proposed above are more universal than Equation (10). First, it does not require additional sensors to be incorporated into the network, and this shall prevail in applications when the sparse transducer array approach is adopted. Moreover, if any of the reference transducers fails or exceeds the bounds of approximation given by the Equation (4), then the damage index obtained for sensing path related to this transducer via formula Equation (10) cannot be compensated. In the alternative compensation methods given by the Equation (13), Equation (14), or Equation (15), it is possible to remove undesirable drift effect, at least partially.

As a number of sensing paths *k* used for damage index drift estimation in the above equations is considered, for properly designed PZT networks, there should exist at least one sensing path emerging from every PZT sensor which is not influenced by damage, unless damage occurs directly in the area where the sensor is located; therefore, k=1 is, in general, a safe assumption. The upper limit for *k* is dependent on network geometry as well as expected localization and damage extent, since if a significant number of sensing paths influenced by damage are included in sets $M_{k}\left(g\right)$, $M_{k}\left(s\right)$, or $M_{k}$ used for EOCs influence estimation, then application of the proposed compensation formulas may have a negative impact on damage detection capabilities.

## 3. Experiment Results and Discussion

In this section, the experiment description, as well as a discussion of the efficiency of different approaches to damage index compensations, are presented.

### 3.1. Experimental Setup

**Figure 2:**
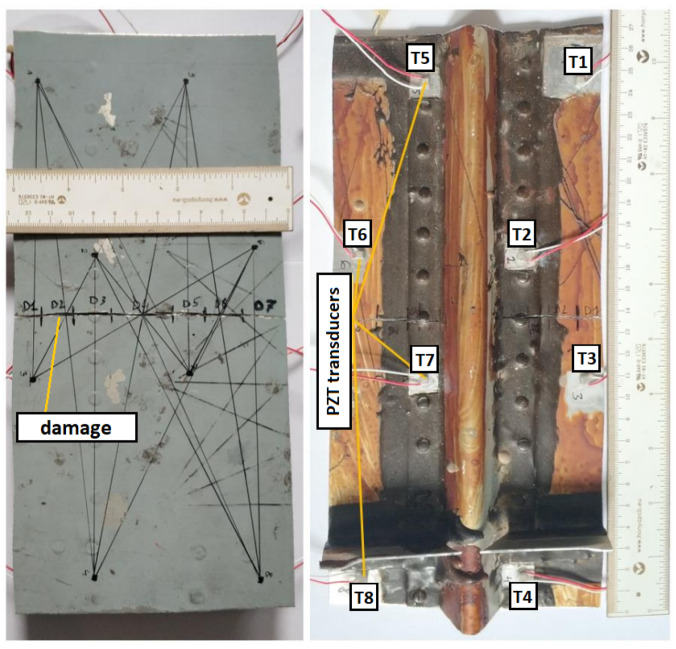
View of specimen no. 1.

**Figure 3:**
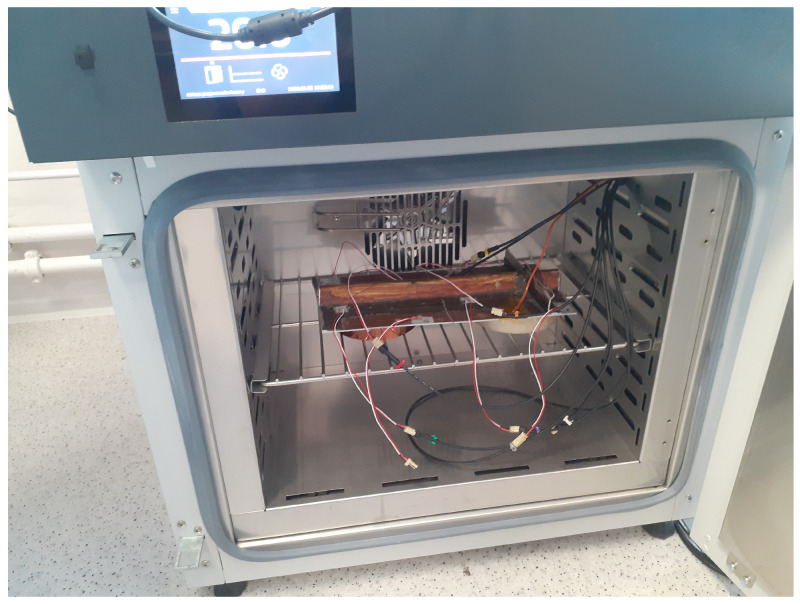
Specimen no. 1 placed in laboratory heater.

In the experiment, two specimens, each equipped with a network of 8 PZT sensors, were used. The first specimen was part of aircraft skin made of aluminum alloy with attached reinforcement (Figure 2). Signals from PZT sensors were acquired for undamaged structure and after damage introduction under varying temperatures in the range 28–63 ${}^{\circ }$C. The introduced damage was a crack machined in the skin part, whereas reinforcement remained intact. The panel was placed in a laboratory heater (Figure 3) during measurements which allowed for homogeneous distribution of temperature across the PZT sensor network.

**Figure 4:**
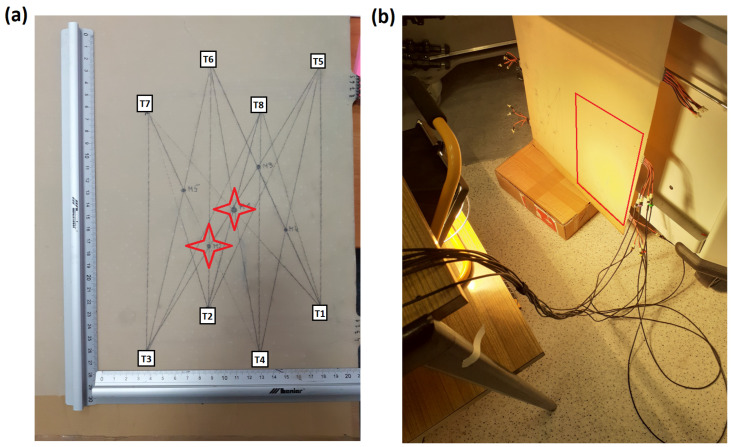
View of specimen no. 2: (**a**) PZT sensors network geometry with an indication of introduced impact damage location; (**b**) specimen heated with the use of halogen lamps with an indication of the monitored area.

**Figure 5:**
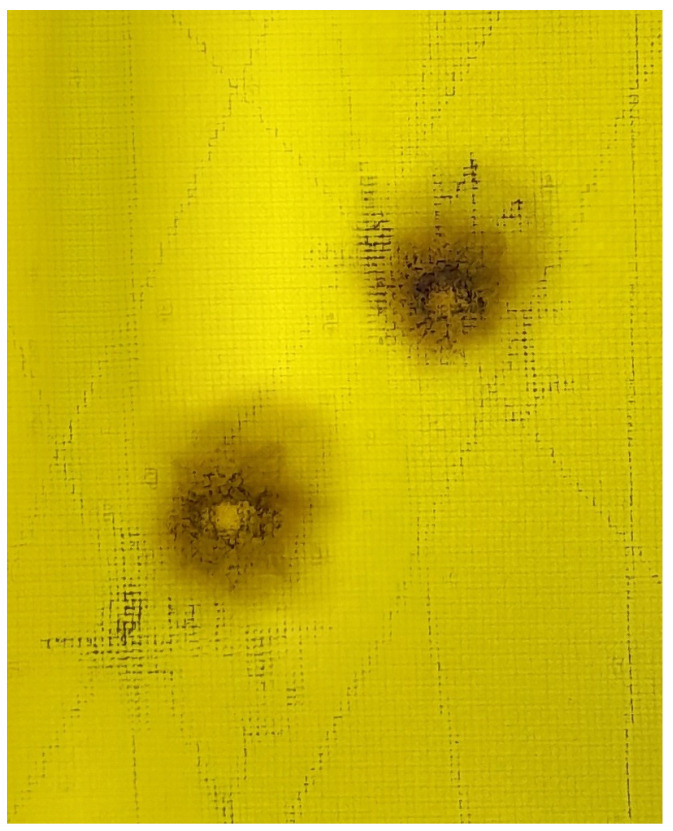
Impact damage introduced in the composite structure.

The second specimen was a part of a GFRP composite panel equipped with a network of 8 PZT sensors transducers embedded into the internal structure of the composite (Figure 4a). Due to the panel dimensions, one or two halogen lamps were used for heat exposure in that case (Figure 4b), and the measurements were performed after thermal balance was established. The temperature of the specimen surface was measured with the use of a non-contact IR Thermometer VIR50 by Extech Instruments. The central point of the PZT network was used as a reference point for temperature measurements. Signals from PZT sensors were acquired under three thermal conditions: at room temperature (homogeneous condition), at a surface temperature of about 45 ${}^{\circ }$C (one halogen lamp used), and at about 65 ${}^{\circ }$C, as measured in the reference point of the specimen. The temperature on the specimen surface varied in the range of ±5 ${}^{\circ }$C under one halogen lamp exposure and ±10 ${}^{\circ }$C when two halogen lamps were used. Two impact damage were introduced in the position indicated in Figure 4a. For that purpose, an air gun able to provide not more than 17 J of kinetic energy to the pellet with an initial speed not higher than 300 m/s was used. The specimen was subjected to two impacts which caused Barely Visible Impact Damage (BVID) as shown in Figure 5.

In the case of the second specimen temperature range used in the study was defined by the experimental setup. Halogen lamps were placed at a safe distance from the specimen, and the temperature was measured after heat transfer was stabilized under exposure to one or two heat sources. The temperature range used for specimen no. 1 was adjusted, respectively. The temperature span, i.e., above 30 ${}^{\circ }$C, should be sufficiently wide for baseline signals collection during system calibration. If the temperature of the monitored object cannot be controlled precisely during measurements, for many applications, the temperature fluctuations should not exceed this level, as the data acquisition process from the PZT network is relatively fast—in this study, collection of signals from the entire network took less than 10 min. Nevertheless, the EOCs compensation method, including the presented one, should be verified in the relevant environment within the expected range of parameters variation individually for a specific application, for example, as a part of the SHM system certification process.

Both PZT networks were composed of 8 PZT sensors. In the case of specimen no. 1, multilayered PZT sensors of the type NAC2002 manufactured by Noliac A/S were used [41]. The sensors were attached to the surface of the specimen, both on the skin part as well as on the reinforcement (Figure 2). For specimen no. 2, single-layered PZT transducers produced by STEMINC (mod. SMD05T04R111WL) were applied [42]. The sensors were embedded into the internal structure of the composite panel in its symmetry plane. For sensors excitation and signal acquisition, PAQ16000D manufactured by EC Electronics (Poland) has been used [43]. As the excitation signal, Hanning windowed, 3-period sine signal at 150 kHz frequency was used in both cases.

### 3.2. Results and Discussion

In this paper, the following damage indices are considered for structure assessment:

(16) $$corr=1-r_{f_{gs},f_{gs,b}},\;divAmp=\left|\log\left(\frac{\max_{t\in T}\left|f_{gs,b}\left(t\right)\right|}{\max_{t\in T}\left|f_{gs}\left(t\right)\right|}\right)\right|$$

where $f_{gs}$, $f_{gs,b}$ denotes the acquired signal and its corresponding baseline, and $r_{f_{gs},f_{gs,b}}$ denotes correlation coefficient between signal and baseline and their envelopes, respectively. For simplicity, it has been assumed above that the average values of signals are negligible:

(17) $$\int_{T}f_{gs}dt\approx 0,\;\int_{T}f_{gs,b}dt\approx 0.$$

The presented damage indicators remain substantially different parts of the information about the details of acquired signals, e.g., divAmp is sensitive only to global amplitude change of the signal, and it reduces information content carried by the signal to its single value, whereas corr damage index is sensitive both to local amplitude changes as well local phase changes of the acquired signals. Before application of compensation formulas, strict symmetry on DIs matrix was imposed by the following formula:

(18) DI(g,s)↦min(DI(g,s),DI(s,g)),

therefore the symmetry constraint recalled in the Equation (6) is represented faithfully. For calculation of compensated damage indices, the corresponding sets $M_{k}\left(g\right)$, $M_{k}\left(s\right)$, $M_{k}$, for k=1,2,3 were used, as defined in Equations (13)–(15). For both cases, k=3 was the upper limit providing that those sets may not contain DIs obtained for sensing paths transversal to damage for all sensors of the network (Figure 2 and Figure 4a).

**Figure 6:**
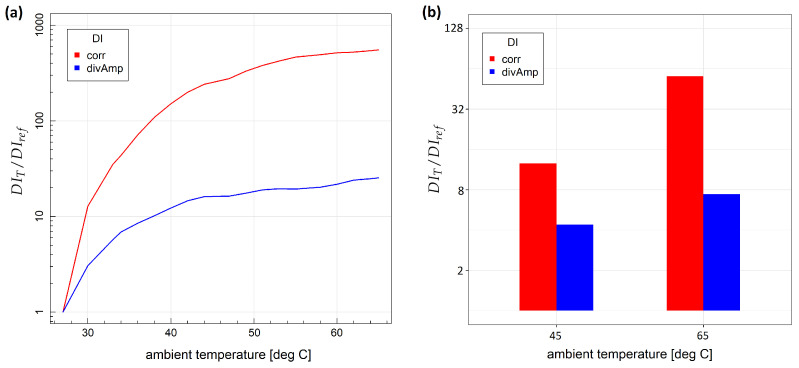
Variability of damage indices with respect to temperature change: (**a**) specimen no. 1; (**b**) specimen no. 2.

**Figure 7:**
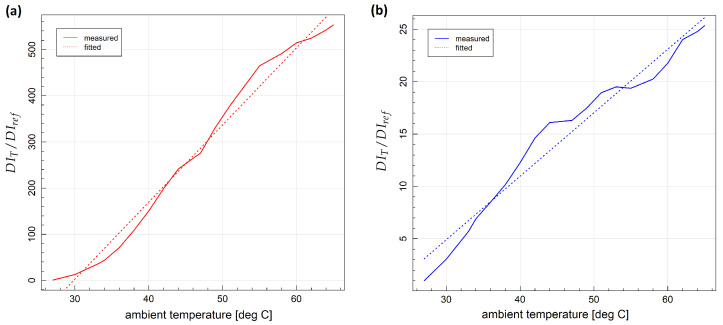
Dependence of relative change of damage indices on temperature for specimen no. 1: (**a**) corr damage index; (**b**) divAmp damage index.

**Figure 8:**
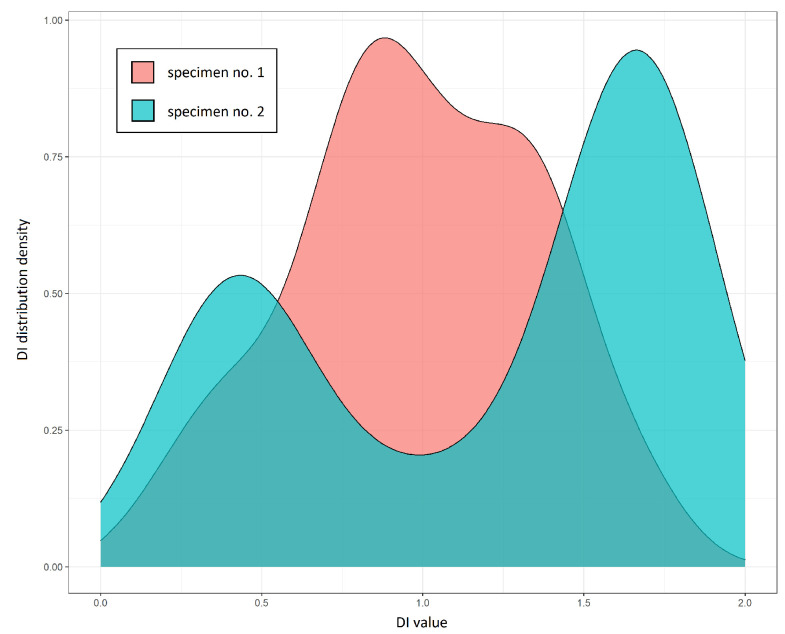
Distribution of corr damage index for the two specimens at a temperature above 60 ${}^{\circ }$C without damage presence.

Variability of the defined damage indices with respect to the temperature for two specimens is shown in Figure 6. The relative change of a given damage index with respect to reference damage index was used as temperature DIs variability measure:

(19) $$DI_{T}/DI_{ref}$$

where $DI_{T}$ denotes the mean of DI values obtained at a given temperature *T* and $DI_{ref}$ is the mean DI value obtained under measurement repeatability condition at the initial temperature. In both cases influence of temperature was greater for corr damage index, as it depends on more details of the signal than divAmp, in particular, on local signal energy distribution, which is reported to be particularly sensitive to temperature variation due to induced wave velocity, piezoelectric parameters and attenuation changes [21]. For metallic structures, the highest relative ratio of temperature-induced DI change was above 500 for the corr damage index and above 20 for the divAmp damage index (Figure 6a). In the case of composite specimens, the highest ratio of DI change due to temperature was above 50 for corr DI and about 8 for divAmp DI (Figure 6b). Relatively small discrepancies of damage indices dependence on temperature from the linear model were observed in the temperature range used in the study (Figure 7), which was one of the assumptions in the derivation of compensated damage index formula based on reference sensing paths (Equation (10)). In Figure 8, the distribution of corr damage index at a temperature above 60 ${}^{\circ }$C without damage presence is shown for both specimens. In the case of specimen no. 1, temperature distribution was homogeneous over sensors of the network; therefore, corr damage index density is unimodal. In the case of specimen no. 2, where two halogen lamps were used for heating, damage index distribution is bimodal with a higher spread than in the case of homogeneous heating.

**Figure 9:**
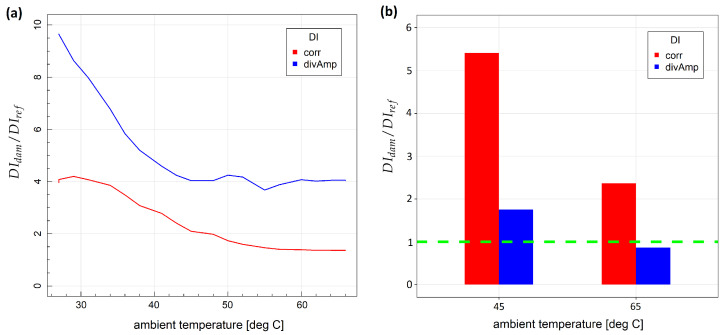
Comparison of damage indices obtained for sensing paths influenced by damage and distant from it under temperature variation: (**a**) specimen no. 1; (**b**) specimen no. 2.

In Figure 9, the damage detection efficiency ratio is

(20) $$D_{eff}=DI_{dam}/DI_{ref},$$

where:

- $DI_{dam}$ denotes the mean value of a given damage index obtained for sensing paths running in the proximity of introduced flaw, which can be sensitive to the transmission mode of guided waves interaction with damage;
- $DI_{ref}$ denotes the mean value of a given damage index obtained for sensing paths running at a significant distance from the introduced damage and thus should be less sensitive to its presence.

In the case of specimen no. 1, the following sensing paths were used in the study:

- to $DI_{dam}$ contributed the following sensing paths: 2–3, 2–7, 3–6, 6–7, 1–3, 1–7, 5–7, 3–5, 2–4, 2–8, 4–6, 6–8;
- to $DI_{ref}$ contributed the following sensing paths: 1–5, 1–6, 1–2, 2–5, 2–6, 5–6, 3–4, 3–7, 3–8, 4–7, 4–8, 7–8.

Other sensing paths of the network were not included in the analysis, as amplitudes of the acquired signals were relatively low. For specimen no. 2:

- to $DI_{dam}$ contributed the following sensing paths: 2–8, 2–5, 4–6, 3–5, 1–7, 1–6, 1–8, 2–6, 2–7, 3–8, 3–6, 4–5, 4–7, 4–8;
- to $DI_{ref}$ contributed the following sensing paths: 1–2, 1–3, 1–4, 2–3, 2–4, 3–4, 5–6, 5–7, 5–8, 6–7, 6–8, 7–8.

The crack introduced in specimen no. 1 partially suppressed the effect of elastic wave transmission between PZT transducers located on opposite sides of the damage, which significantly affected the global amplitude of the acquired signals. Therefore, the divAmp damage index was significantly more efficient than the corr damage index in damage detection in that case. For divAmp characteristic damage efficiency ratio was nearly 10 under measurement repeatability conditions (Figure 9a) and about 4 at the highest temperature. The efficiency of the corr damage index in damage detection, in that case, was about 55% lower on average. In the case of BVID damage introduced in specimen no. 2, the corr damage index exhibited higher efficiency in damage detection than the divAmp damage index. At elevated temperatures, $D_{eff}$ coefficient obtained for corr damage index was significantly greater than that obtained for divAmp damage index. At the highest temperature, the divAmp signal characteristic did not allow for distinction between sensing paths close to damage and reference sensing paths; therefore, it was not possible to detect damage based on its values, whereas for corr coefficient $D_{eff}$ was greater than 2.

**Figure 10:**
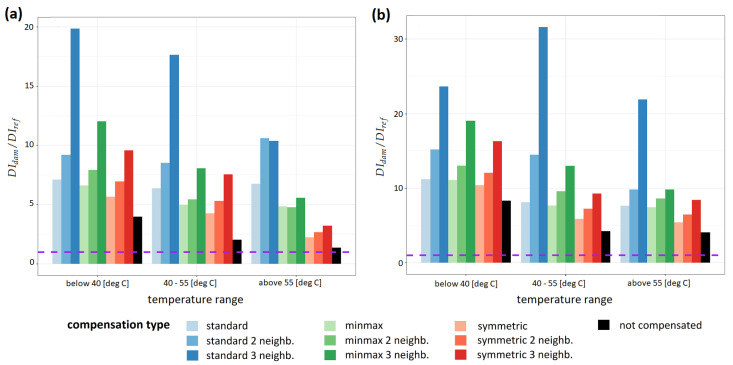
Comparison of damage detection efficiency for different schemes of damage indices compensation at different temperature levels for specimen no. 1: (**a**) for corr damage index; (**b**) for divAmp damage index.

**Figure 11:**
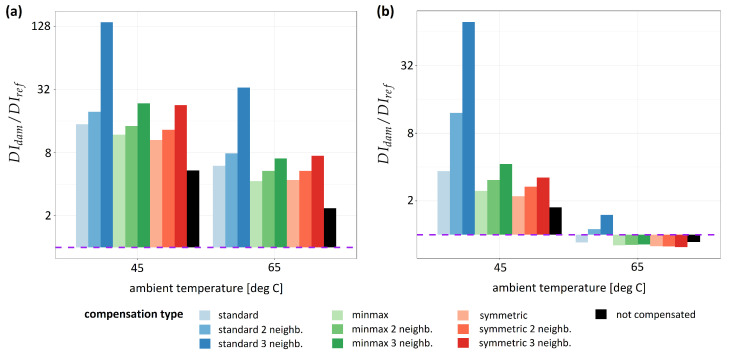
Comparison of damage detection efficiency of different schemes of damage indices compensation at different temperature levels for specimen no. 2: (**a**) for corr damage index; (**b**) for divAmp damage index.

In Figure 10 and Figure 11, damage detection efficiency ratio $D_{eff}$, defined by the Equation (20) and obtained for different schemes of damage indices compensation, is shown for specimen no. 1 and specimen no. 2, respectively. For specimen no. 1 (Figure 10) median values of $D_{eff}$ obtained for indicated temperature range are shown. The linear scale was applied for the presentation of the results obtained for specimen no. 1 (Figure 10), in the case of specimen no. 2 (Figure 11) logarithmic scale was used. The dashed line in both plots represents $D_{eff}=1$, which is a limiting value for the possibility of distinction of sensing paths influenced by damage and thus SHM system applicability. For all compensation schemes, the ratio $D_{eff}$ in most cases is higher if the compensation formula is used. The best damage detection capability was obtained for the standard compensation procedure given by the Equation (13), irrespectively of DIs or damage type as well as temperature range. Moreover, damage detection efficiency $D_{eff}$ is enhanced if more sensing paths are included in sets $M_{k}$ used for estimation of Damage Indices drift effects, the best results were observed for k=3 for all compensation formulas. For corr damage index $D_{eff}$ coefficient was about 10 for specimen no. 1 at temperatures above 55 ${}^{\circ }$C (Table 1) and about 33 for specimen no. 2 at temperatures about 65 ${}^{\circ }$C (Table 2), and it was, respectively, about 750% and 1400% higher than $D_{eff}$ obtained for uncompensated damage index. For divAmp damage index damage detection efficiency coefficient at the highest temperatures was about 22 for specimen no. 1, which was 540% higher than $D_{eff}$ obtained for uncompensated damage index (Table 3). For divAmp damage index obtained for specimen no. 2 at the highest temperature (Figure 11b, Table 4) $D_{eff}$ coefficient was about or below 1, except for standard compensation scheme with k=3 for which $D_{eff}$ obtained was about 1.5. This means that without the application of proper compensation formula, the damage could not be detected, as the distinction between sensing paths close to damage and reference sensing paths wouldn’t be possible.

**Figure 12:**
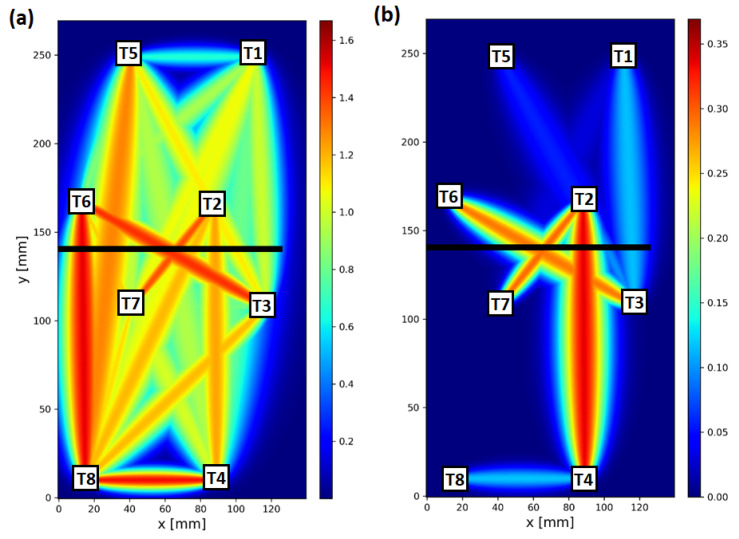
Visualization of corr damage index for specimen no. 1 above 60 ${}^{\circ }$C with an indication of introduced damage and sensors localization: (**a**) uncompensated damage index; (**b**) standard compensation scheme with k=3.

**Figure 13:**
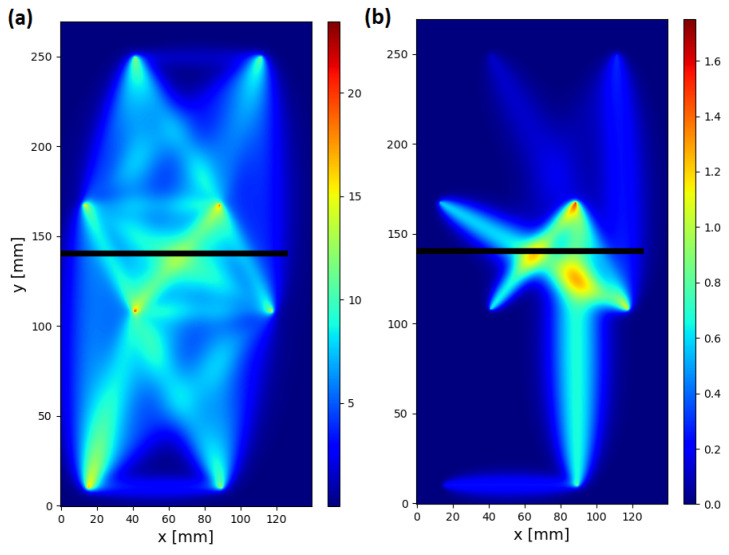
Averaged visualization map of corr damage index for specimen no. 1 above 60 ${}^{\circ }$C with indication of introduced damage: (**a**) uncompensated damage index; (**b**) standard compensation scheme with k=3.

In Figure 12, visualization of raw and compensated corr damage index values within the network for specimen no. 1 above 60 ${}^{\circ }$C is shown. In both cases, the colormap was automatically adjusted to be 10% higher than the maximum value of DIs obtained within the network. As the compensation formula (Equation (13)) reduces DIs values for all sensing paths of the network, the use of individually adopted colormaps is legitimate, since also the threshold for damage indication should be adjusted accordingly for structure assessment based on compensated DIs. Before compensation, sensing paths not sensitive to the transmission mode of elastic wave interaction with introduced damage, for which significant corr value was obtained due to temperature influence, were as follows (Figure 12a): 1–5, 2–5, 3–8, 4–7, 4–8, 7–8. In particular, the corr value for sensing path 4–8, located at a significant distance from damage, was comparable to the DI value obtained for sensing paths transversal to damage. After compensation (Figure 12b), from the mentioned sensing paths, a significant corr value was obtained only for sensing path 4–8, so the temperature influence was still significant in this case. In Figure 13, averaged visualization maps, based on RAPID imaging algorithm [38,39], obtained for raw and compensated data are presented. Application of the proposed compensation formula allows not only for the improvement of contrast between damage-influenced and reference sensing paths but also provides a better basis for damage localization. Averaged damage intensity map obtained for compensated coor index is more confined to the damaged region of the specimen (Figure 13b) than in the case of raw DI (Figure 13a).

## 4. Summary

In the paper, three different damage index compensation formulas have been proposed, and their efficiency with respect to temperature variation was investigated both for uniform as well as non-homogeneous temperature distribution over the PZT network. The method was applied to the compensation of damage indices carrying different information content of signals acquired by PZT sensors. Moreover, different types and extents of damage were investigated. It was shown in the paper, that application of damage compensation formulas can significantly enhance the damage detection efficiency of damage indices. In the best-case scenario, the efficiency of the damage index increased by over 1400%. Application of the proposed compensation formula allows not only for the improvement of contrast between damage-influenced and reference sensing paths, i.e., damage detection efficiency, but also provides a better basis for damage localization.

It is worth noticing again that despite the proposed method being verified in the case of temperature variation, it can also be applied in general cases. The EOCs driving undesirable effects on damage indices neither need to be measured nor have to be known. It is the damage index itself which, through its values obtained over all sensing paths of the network, carries joint information about factors influencing measurement outcome, and a proper combination of damage index values is used for compensation. Nevertheless, further studies are required for proper efficiency assessment of the presented algorithms with respect to other EOCs compensation.

## Figures and Tables

**Table 1:** $D_{eff}$ coefficient for corr damage index obtained for specimen no. 1.

		Temperature Range		
		**below 40** ${}^{\circ }$ **C**	**40** ${}^{\circ }$ **C–55** ${}^{\circ }$ **C**	**above 55** ${}^{\circ }$ **C**
**compensation scheme**	standard k=1	7.11	6.37	6.76
	standard k=2	9.21	8.53	10.59
	standard k=3	19.85	17.62	10.36
	minmax k=1	6.62	4.97	4.84
	minmax k=2	7.93	5.43	4.76
	minmax k=3	12.02	8.07	5.56
	symmetric k=1	5.65	4.26	2.25
	symmetric k=2	6.95	5.31	2.65
	symmetric k=3	9.59	7.55	3.20
	not compensated	3.97	2.04	1.37

**Table 2:** $D_{eff}$ coefficient for corr damage index obtained for specimen no. 2 (temperature measured at the reference point of the surface).

		Temperature	
		**45** ${}^{\circ }$ **C**	**65** ${}^{\circ }$ **C**
**compensation scheme**	standard k=1	14.91	5.99
	standard k=2	19.60	7.87
	standard k=3	139.15	33.34
	minmax k=1	11.84	4.28
	minmax k=2	14.30	5.35
	minmax k=3	23.53	14.00
	symmetric k=1	10.55	4.37
	symmetric k=2	13.20	5.37
	symmetric k=3	22.63	7.45
	not compensated	5.41	2.37

**Table 3:** $D_{eff}$ coefficient for divAmp damage index obtained for specimen no. 1.

		Temperature Range		
		**below 40** ${}^{\circ }$ **C**	**40** ${}^{\circ }$ **C–55** ${}^{\circ }$ **C**	**above 55** ${}^{\circ }$ **C**
**compensation scheme**	standard k=1	11.22	8.10	7.64
	standard k=2	15.18	14.47	9.82
	standard k=3	23.63	31.60	21.87
	minmax k=1	11.11	7.66	7.42
	minmax k=2	12.99	9.59	8.61
	minmax k=3	19.05	12.96	9.82
	symmetric k=1	10.40	5.85	5.39
	symmetric k=2	12.02	7.23	6.45
	symmetric k=3	16.30	9.28	8.42
	not compensated	8.31	4.21	4.04

**Table 4:** $D_{eff}$ coefficient for divAmp damage index obtained for specimen no. 2 (temperature measured at the reference point of the surface).

		Temperature	
		**45** ${}^{\circ }$ **C**	**65** ${}^{\circ }$ **C**
**compensation scheme**	standard k=1	3.68	0.85
	standard k=2	12.14	1.12
	standard k=3	78.10	1.50
	minmax k=1	2.44	0.80
	minmax k=2	3.06	0.81
	minmax k=3	4.26	0.82
	symmetric k=1	2.20	0.79
	symmetric k=2	2.68	0.79
	symmetric k=3	3.22	0.78
	not compensated	1.75	0.86

## Data Availability

Data used in this study are available on-demand from the corresponding author.

## Abbreviations

The following abbreviations are used in this manuscript:

BVID	Barely Visible Impact Damage
DI (DIs)	damage index (damage indices)
EOC (EOCs)	environmental and operational condition (conditions)
GFRP	Glass Fiber Reinforced Polymer
SHM	structural health monitoring
